# Center Volume Not Associated with Survival Benefit of Inter-hospital Transfer for Pediatric CardiacSurger

**DOI:** 10.21203/rs.3.rs-5356715/v1

**Published:** 2024-11-20

**Authors:** Dhaval Chauhan, J. Hunter Mehaffey, J. W. Awori Hayanga, Pieter Alex Verhoeven, Margaret Mathewson, Veronica Godsey, Alyssa Fazi, Jai P. Udassi, Vinay Badhwar, Christopher E. Mascio

**Affiliations:** West Virginia University; West Virginia University; West Virginia University; West Virginia University; West Virginia University; West Virginia University; West Virginia University; West Virginia University; West Virginia University; West Virginia University

**Keywords:** Hospital transfer, annual volume, pediatric cardiac surgery, mortality

## Abstract

**Objective:**

To evaluate the relationship between center volume and inpatient mortality after inter-hospital transfer among patients undergoing pediatric cardiac surgery using contemporary real-world data.

**Methods:**

The Kids’ Inpatient Database (KID) was queried for cardiopulmonary bypass (CPB) cases (CPB) for years 2016 and 2019. Hospitals were divided into three groups based on terciles of volume: “low”: ≤103 cases/year, “mid”:104–194 cases/year, and “high”: >194 cases/year. Multilevel regression models were created to evaluate the association of volume and inpatient mortality for transferred patients for the entire cohort as well as high-complexity cases. (Risk Stratification for Congenital Heart Surgery (RACHS-2) categories 3,4 and 5)

**Results:**

Of 25,749 patients undergoing cases on CPB, 3,511 (13.6%) were preoperative inpatient transfers between hospitals. Compared to direct admissions, unadjusted mortality for patients who were transferred was higher in all groups: 1.7% vs. 5.6% (low-volume), 1.1% vs. 4.6% (mid-volume) and 1.1% vs. 4.9% (high-volume). Compared to low-volume hospitals, inpatient mortality for patients admitted on transfer was not significantly different in mid-volume (OR = 0.85, 95% CI 0.54–1.34, p = 0.483) and high-volume centers (OR = 0.7, 95% CI 0.45–1.12, p = 0.127) for the entire cohort. There was no significant difference in risk-adjusted inpatient mortality for high-complexity cases performed at mid-volume (OR 1.06, p = 0.845, 95% CI (0.62–1.85)) or high-volume hospitals (OR 0.82, p = 0.482, 95% CI (0.48–1.45)).

**Conclusion:**

Annual CPB case volume may not accurately predict risk-adjusted inpatient mortality for children transferred for heart surgery. Annual case volume alone should not dictate transfer practices in pediatric heart surgery.

## Introduction

Contemporary reports within the literature suggest that higher volume is associated with improved outcomes in patients undergoing pediatric cardiac surgery. [1–7] However, volume alone is an imperfect metric to assess quality. Although a volume-outcome relationship may exist, it has been observed that high quality is present in centers of all volume levels. [8, 9] Overall outcomes after pediatric cardiac surgery are excellent and are likely attributable to combination of factors including improved surgical techniques and standardized intensive care unit (ICU) care. [10–16]

Recent expert consensus recommendations suggest the creation of a tiered system for pediatric cardiac surgery centers in the United States and go as far as to suggest that neonatal and high-complexity surgeries should not be performed in low-volume centers. [17] The implementation of such a tiered system may limit access to care and potentially result in the initiation of transfers solely based on differences in annual volume, without more objective correlation to the quality of care provided. This practice may result in increased cost, inefficiency and risks overlooking the utility and pragmatism of providing care lower volume but potentially high-quality center.

To date, there is paucity of literature that investigates the outcomes of patients undergoing inter-hospital transfer for the purpose of pediatric cardiac surgery. Further there is little evidence to support the volume-outcome associations in this subset of patients. Therefore, the goal of this study was to explore the association of hospital volume and inpatient mortality in pediatric patients transferred for cardiac surgery using a national cohort of real-world contemporary data. We hypothesized that there would be no difference in inpatient risk-adjusted all-cause mortality and incidence of post-operative ECMO in patients undergoing pediatric cardiac surgery among hospitals grouped by their annual case volume.

## Methods

### Data source and inclusion criteria:

Data from the Kids’ Inpatient Database (KID) for years 2016 and 2019 were utilized for this retrospective cohort analysis. KID belongs to the Healthcare Cost and Utilization Project (HCUP). and is the largest publicly available all-payer claims-based pediatric database. KID is an administrative database. This database samples up to 80% of complex newborn and pediatric discharges (< 21 years) from forty-nine US states. [18]

The pertinent congenital heart disease diagnoses and pediatric cardiac surgical procedures were derived using the International Classification of Diseases, 10th Edition, Clinical Modification (ICD-10-CM) and Procedure Coding System (ICD-10-PCS) respectively. The study included pediatric patients (< 21 years) undergoing cardiac surgery under cardiopulmonary bypass (CPB). Only the hospitals performing at least one of the ten STS benchmark procedures were included in the study. Patients were extracted from the database using the algorithm published by Allen et al. [19] Patients being transferred for the surgery from another hospital were extracted as a subgroup. Patients from the hospitals only performing ventricular septal defect as a surgery were excluded as well as those undergoing off-pump coarctation from thoracotomy. The off-pump cases e.g. delayed chest closures, explorations for bleeding, band adjustments, washouts for infections, and ECMO cannulation were excluded from the analysis. Patients undergoing thoracic organ transplantation or ventricular assist device placement were also excluded.

The West Virginia University Institutional Review Board approved the study with waiver of consent (Protocol #2210660362, approved 3/27/2023).

### Statistical methods

Based on total annual number of CPB cases, hospitals were divided into three volume-groups: low, mid, and high-volume hospitals. This was done based on annual volume terciles. The baseline characteristics were compared using chi-square tests and one way analysis of variance (ANOVA) tests. Primary outcome measure was risk-adjusted all-cause inpatient mortality. The secondary measure was risk-adjusted incidence of post-operative extracorporeal membrane oxygenation (ECMO). Multi-level multivariable logistic regression was used for the transfer cohort and high complexity transfers subgroup (Risk Stratification for Congenital Heart Surgery (RACHS-2) category 3, 4, and 5 cases) to investigate the association of center volume and risk-adjusted inpatient mortality and post-operative ECMO. Using the same model, predicted risk-adjusted mortality was derived for each hospital in the study. Separate multi-level models were created for transferred patients to investigate impact of center volume on outcomes for each individual RACHS-2 category. A brief description of multilevel analysis is included in paragraph 1 of the supplementary appendix. Histograms were plotted for predicted inpatient mortality by hospital volume groups for all transfer patients, for high-complexity transfer cases and by each RACHS-2 category transfer cases.

### Risk-adjustment

The algorithm for risk stratification for pediatric cardiac surgery by surgical procedure/complexity for administrative data published by Allen et al. was used to calculate the RACHS-2 score for all patients. [19] This algorithm has been verified for its accuracy and validated for the KID database. [20] RACHS-2 was added as an ordinal variable in the regression analysis. Hospital volume was included as an independent variable comparing low-volume centers directly to mid-volume and high-volume centers. Patient age, gender, race, elective status, genetic diagnosis, reoperation, and preoperative length of stay were added as covariables. These variables were chosen based on the previously published literature. [21–24] Please see supplemental table 1 for details of ICD-10 codes used to derive key covariables.

Missing data was handled by imputing the missing variables by the Multiple Imputation by Chained Equations (MICE) method. [25, 26] Regression models were investigated for lack of collinearity between covariables. A p-value of less than 0.05 was considered as a statistically significant value. All tests were two-sided. Statistical package R was used for statistical analysis. [27]

## Results

Of the 25,749 children undergoing CPB, 3,511 (13.66%) patients were transferred to the hospital where they received surgery from another hospital. The center volume categories were distributed as follows: 113 low-volume hospitals performing ≤ 103 cases per year; 61 mid-volume hospitals performing 104–194 cases per year; and 31 high-volume hospitals performing > 194 cases per year. The median (interquartile range) number of transferred cases per hospital per year were 4.5 (2–9) for low-volume hospitals, 21 (11–31) for mid-volume hospital and 43 (34–61) for high-volume hospitals. The distribution of STS benchmark procedures by hospital volume groups is displayed in supplemental table 2. The distribution of cases by each hospital is shown in supplemental Fig. 1.

Two-thirds of the transferred patients were neonates. As expected, more than 90% of the procedures performed were urgent or emergent procedures. Low-volume centers had higher number of patients with RACHS-2 category 1 patients and lower number of RACHS-2 category 5 patients compared to mid and high-volume centers. (Table 1).

The unadjusted mortality rate for the entire transfer cohort was 5.2%. The unadjusted inpatient mortality rates for low, mid and high-volume centers were 5.6%, 4.6% and 4.9% respectively. For direct admissions, these rates were 1.7%, 1.13% and 1.1% respectively.

### Results for all transferred patients

**Figure 1:**
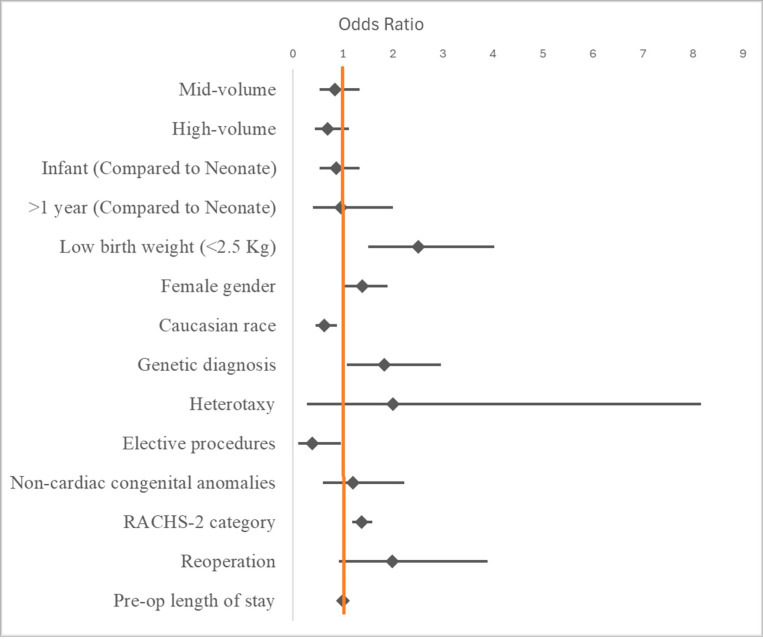
Forest plot showing Odds ratios of variables included in the multivariable logistic regression model for inpatient mortality.

The risk-adjusted inpatient mortality rate for all transferred patients was 4.99%. The risk-adjusted inpatient mortality rates for low, mid and high-volume centers were 5.4%, 5.1% and 4.3% respectively. Following multilevel multivariable regression analysis for all transferred patients, there was no statistically significant difference in risk-adjusted inpatient mortality when comparing low-volume centers to mid-volume centers (OR = 0.85, 95% CI 0.54–1.33, p = 0.469) or to high-volume centers (OR = 0.7, 95% CI 0.45–1.12, p = 0.127). Factors associated with increased inpatient mortality included low birth weight, non-Caucasian race, presence of a genetic diagnosis, higher RACHS-2 score, emergent/urgent procedures and reoperation (Fig. 1, Table 2).

**Figure 2:**
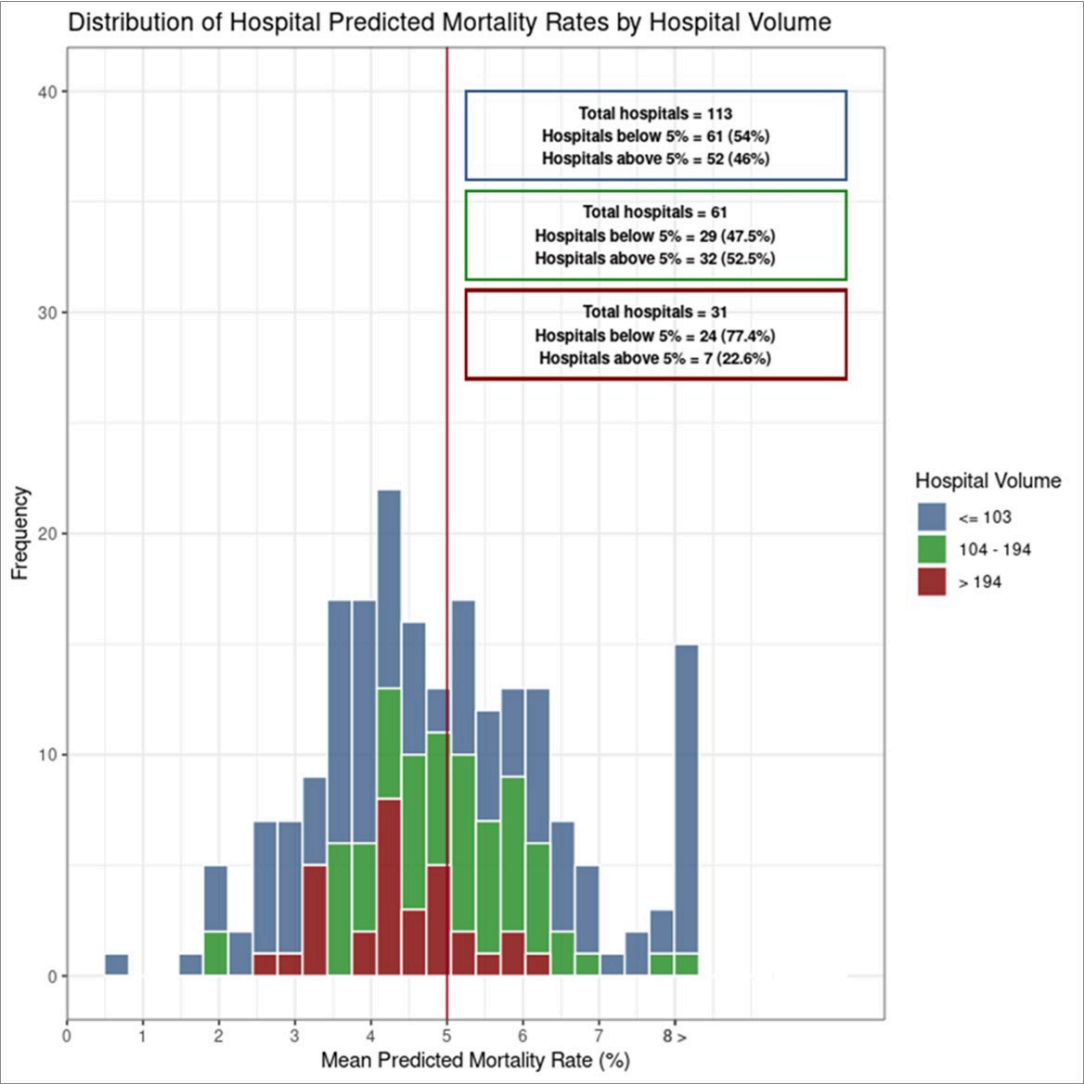
Histogram of risk-adjusted inpatient mortality by hospital volume groups for the entire cohort

The distribution of risk-adjusted mortality was similar across all volume groups (Fig. 2), highlighting low-mortality and high-mortality hospitals in all three volume categories.

The incidence of ECMO was 3.2% within the transferred cohort. It was 4.0% for low-volume centers, 2.8% for mid-volume centers and 3.2% for high-volume centers. There was no statistically significant difference in the incidence of post-operative ECMO between low-volume centers and mid-volume centers (OR = 0.59, 95% CI 0.26–1.39, p = 0.218) and low-volume centers and high-volume centers (OR = 0.74, 95% CI 0.3–1.89, p = 0.502). Factors associated with increased incidence of post-operative ECMO were low birth weight and female gender (Table 3).

### Results for RACHS-2 high-risk category transfer cohort

**Figure 3:**
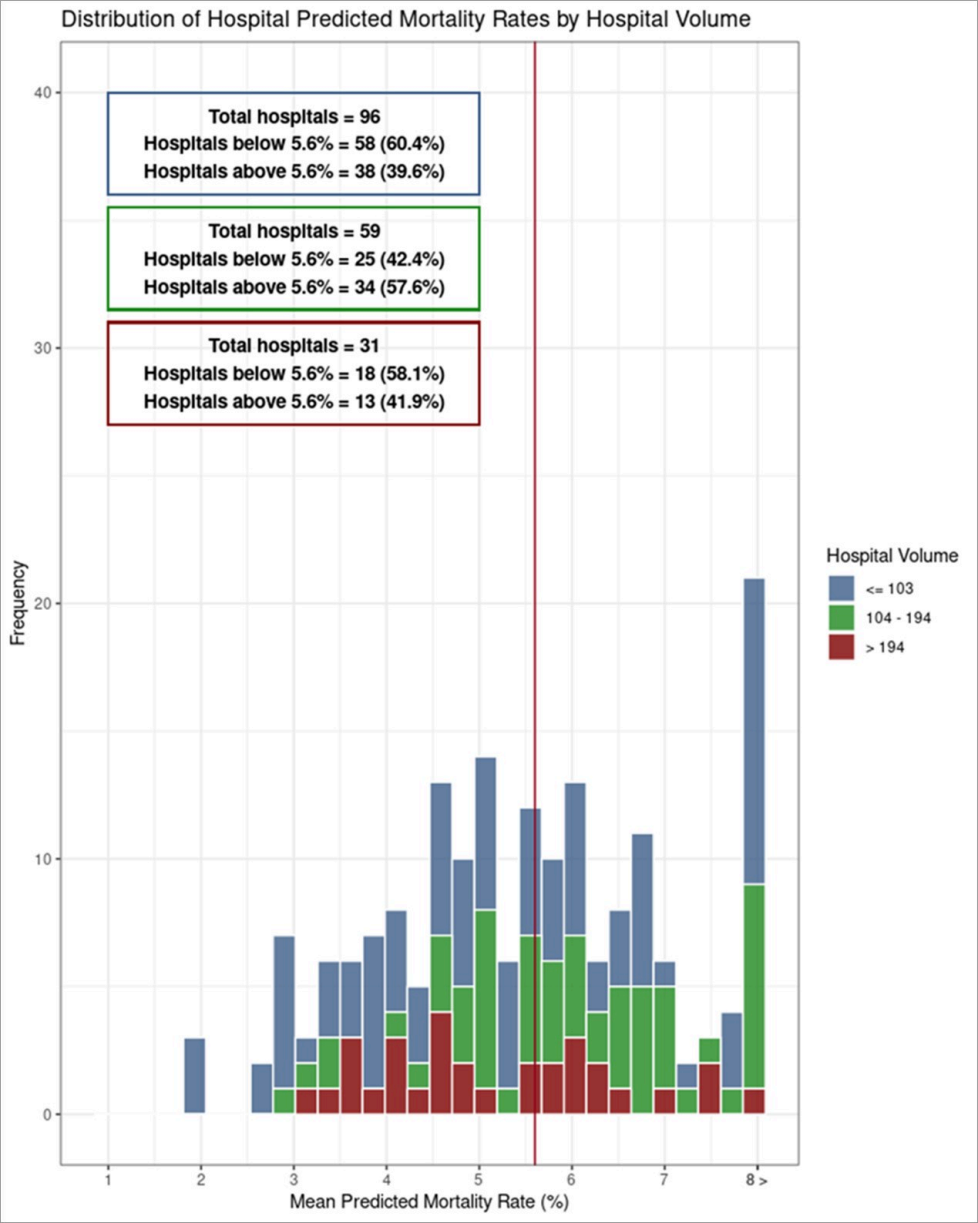
Histogram of risk-adjusted inpatient mortality by hospital volume groups for high-complexity cases (RACHS-2 categories 3, 4 and 5)

The risk-adjusted inpatient mortality rate for high-risk cohort was 5.6%. The risk-adjusted inpatient mortality rates for low, mid and high-volume centers were 5.5%, 6.1% and 5.2% respectively. There was no statistically significant difference in risk-adjusted inpatient mortality for high-complexity transfer cases (RACHS-2 category 3,4 and 5) between low-volume hospitals and mid-volume hospitals (OR = 1.06, 95% CI 0.62–1.85, p = 0.845) and high-volume hospitals (OR = 0.82, 95% CI 0.48–1.45, p = 0.482) (Supplemental table 3). Similarly, we observed low-mortality and high-mortality hospitals in all three hospital groups (Fig. 3).

There was no statistically significant difference in incidence of post-operative ECMO between low-volume and mid-volume hospitals (OR = 0.51, 95% CI 0.21–1.24, p = 0.127) or high-volume hospitals (OR = 0.58, 95% CI 0.24–1.54, p = 0.243). (Supplemental table 4)

The results of multilevel multivariable logistic regression analyses for individual RACHS-2 categories also showed no statistically significant difference in inpatient mortality for transferred patients (Table 4).

Histograms of the independent RACHS-2 categories showed that the hospital volume did not correlate with the risk-adjusted inpatient mortality with mid-volume hospitals having high mortality in RACHS-2 category 1 cases, low-volume hospitals having high mortality in RACHS-2 category 2 cases, and high-volume hospitals having high mortality in RACHS-2 category 3 cases (Supplemental Figs. 2–6).

## Discussion

This study presents a risk-adjusted real-world analysis of patients transferred from one hospital to another for pediatric cardiac surgery with four main findings. First, the overall mortality of the cohort was approximately 5%, highlighting the high-risk nature of this cohort of patients. Second, after risk adjustment, there was no significant difference in inpatient mortality or incidence of ECMO based on hospital case volume. Third, when assessing the high-complexity subgroup (RACHS-2 categories 3–5) hospital case volume did not predict inpatient mortality in transfer cases. Finally, we highlight there are high performing low volume centers and low performing high volume centers when assessing risk-adjusted inpatient mortality by each RACHS-2 category.

Our analysis highlights the fact that hospital quality may be a more important determinant of inpatient mortality or incidence of post-operative ECMO than annual case volume in transferred patients undergoing pediatric heart surgery, even for high-complexity cases and hospital volume may not play a role in it. This is in contradistinction to recent expert consensus for centers performing pediatric cardiac surgery in the US that suggests transfers of neonates and patients requiring complex congenital cardiac surgery should only be to higher volume centers. [17] Access to care for patients with congenital heart disease remains a hurdle in the US, especially in socially disenfranchised populations. [28–30] If a high-quality low-volume center is closer to the patient and caregiver(s), they may not need to travel farther to a higher volume center. This may translate into increased access to care, increased compliance in post-operative follow-up and limited extended caregiver life disruption. This might be particularly beneficial, as they may be spared from having to take time away from their work, ensuring a stable income and normalcy in day-to-day life. In cases where the local center is within the same state as the patient/caregiver(s) residence and the larger center is outside the state, it may also have significant implications in insurance coverage.

These data demonstrate that there are also underperforming high volume centers, suggesting that transfer to larger programs does not uniformly guarantee superior outcomes. These findings align with previous work supporting the creation of a tiered system based solely on annual hospital volume may not necessarily be the correct sole arbiter of quality. ^9,17^ The incidence of mortality in these transferred patients was strikingly high at all recipient volume levels. Although a definite cause cannot be identified through our analysis, the plausible causes may include higher comorbidities, possible errors and delays in diagnosis or management, and a higher possibility of complications at the initial hospital encounter. Most of the transferred patients were neonates, and perhaps some may not have received standard prenatal care, their diagnosis was possibly delayed, or they presented to a hospital without resources to manage complex congenital heart disease.

Our subgroup analysis of hospital volume and inpatient mortality by each RACHS-2 category showed that in each category, there was significant variation and inconsistency without a clear association between higher annual case volume and improved mortality. For the entire cohort and for high-complexity cases, there existed high-mortality and low-mortality hospitals in every volume group. This reveals that higher hospital volume does not guarantee a better result after pediatric cardiac surgery, even when considering the complexity of the heart lesion or the technique of surgery.

There are several important limitations to this analysis including the retrospective nature of the study which precludes demonstration of causality. The administrative data may be subject to errors during coding; however, KID shares a robust quality control audit process with the HCUP family of databases. Regression models are limited to the data available in the database and therefore lack some key variables including surgeon experience, time on cardiopulmonary bypass and other comorbidities that could not be captured by the database. The KID database does not have the information on whether the referring hospital had a congenital heart surgery service or not. Even though in a real-world scenario, majority of the transfers are from hospitals without the surgical services, there might be some patients transferred from a hospital with surgical services for higher level of care. Our hierarchal models and multilevel analysis correct this bias to a certain extent, however without the information about referent hospital origin, it cannot be removed completely. Finally, although our analysis had sufficient statistical power to identify difference in in-patient mortality for the cohorts we reported, results for individual complex operations could not be analyzed due to their small sample size.

## Conclusions

Pediatric patients transferred from one hospital to another for \ cardiac surgery are of higher risk, regardless of the volume of the receiving institution. Hospital quality may be a more important metric than hospital volume to predict inpatient mortality or incidence of post-operative ECMO. Volume of the reference hospital alone should not be considered as the sole decision-making metric to refer patients for congenital heart surgery.

## Supplementary Material

Supplement 1

## Figures and Tables

**Table 1:** Baseline characteristics of the cohort of patients transferred in from another hospital.

Variable	Total (3,511)	Low-volume (669)	Mid-volume (1,266)	High-volume (1,576)	p-value
Age					
Neonate	2,476 (70.5%)	457 (68.3%)	929 (73.4%)	1,090 (69.2%)	0.023
Infants	829 (23.6%)	166 (24.8%)	262 (20.7%)	401 (25.4%)	
> 1 year	206 (5.9%)	46 (6.9%)	75 (5.9%)	85 (5.4%)	
Female gender	1,494 (42.6%)	304 (45.4%)	537 (42.4%)	653 (41.4%)	0.212
Caucasian	1,730 (49.3%)	317 (47.4%)	682 (53.9%)	731 (46.4%)	< 0.001
Low birth weight	205 (5.8%)	37 (5.5%)	70 (5.5%)	98 (6.2%)	0.688
Genetic diagnosis	234 (6.7%)	34 (5.1%)	82 (6.5%)	118 (7.5%)	0.106
Heterotaxy	15 (0.4%)	1 (0.1%)	8 (0.6%)	6 (0.4%)	0.281
Elective procedures	197 (5.6%)	41 (6.13%)	50 (3.9%)	106 (6.7%)	0.005
Non-cardiac congenital anomalies	170 (4.8%)	27 (4.0%)	69 (5.5%)	74 (4.7%)	0.362
RACHS-2 score					
1	377 (10.7%)	92 (13.8%)	127 (10%)	158 (10%)	<0.001
2	928 (26.4%)	201 (30%)	340 (26.9%)	387 (24.6%)	
3	543 (15.5%)	117 (17.5%)	188 (14.8%)	238 (15.1%)	
4	1,134 (32.3%)	206 (30.8%)	400 (31.6%)	528 (33.5%)	
5	529 (15.1%)	53 (7.9%)	211 (16.7%)	265 (16.8%)	
Reoperation	136 (3.9%)	14 (2.1%)	48 (3.8%)	74 (4.7%)	0.014
Pre-operative length of stay	9.26 (± 16.3)	11.1 (± 20)	9.6 (± 15.3)	8.19 (± 15.2)	0.001

**Table 2:** Results of multi-level logistic regression models for inpatient mortality as outcome for the entire cohort

Variable	OR (95% CI)	p-value
Hospital volume		
Low-volume	1	(reference)
Mid-volume	0.85 (0.54–1.34)	0.483
High-volume	0.7 (0.45–1.12)	0.127
Age		
Neonate	1	(reference)
Infant	0.87 (0.54–1.34)	0.535
>1 year	0.96 (0.4–2)	0.913
Low birth weight (< 2.5 kg)	2.51 (1.51–4.03)	< 0.001
Female gender	1.39 (1.01–1.9)	0.04
Caucasian race	0.63 (0.46–0.88)	0.006
Genetic diagnosis	1.83 (1.08–2.96)	0.019
Heterotaxy	2.01 (0.29–8.16)	0.388
Elective procedures	0.39 (0.11–0.96)	0.071
Non-cardiac congenital anomalies	1.21 (0.6–2.23)	0.557
RACHS-2 category	1.38 (1.19–1.59)	0.001
Reoperation	1.99 (0.93–3.89)	0.056
Pre-op length of stay	1.01 (1–1.02)	0.001

RACHS-2: Risk Adjustment for Congenital Heart Surgery

**Table 3:** Results of multi-level logistic regression models for post-operative ECMO as outcome for the entire cohort

Variable	OR (95% CI)	p-value
Hospital volume		
Low-volume	1	(reference)
Mid-volume	0.66 (0.28–1.64)	0.358
High-volume	0.8 (0.32–2.15)	0.632
Age		
Neonate	1	(reference)
Infant	0.49 (0.25–0.91)	0.029
>1 year	0.53 (0.15–1.47)	0.274
Low birth weight (< 2.5 kg)	1.87 (0.88–3.69)	0.083
Female gender	1.34 (0.88–2.03)	0.171
Caucasian race	0.75 (0.48–1.16)	0.197
Genetic diagnosis	1.19 (0.53–2.4)	0.64
Heterotaxy	10.83 (1.84–52.67)	0.004
Elective procedures	0.95 (0.35–2.21)	0.918
Non-cardiac congenital anomalies	1.3 (0.51–2.87)	0.551
RACHS-2 category	1.25 (1.04–1.51)	0.021
Reoperation	1.84 (0.65–4.44)	0.205
Pre-op length of stay	1.01 (1–1.02)	0.191

**Table 4:** Results of multilevel multivariable logistic regression models for risk-adjusted inpatient mortality by each RACHS-2 category

RACHS-2 category	Hospital volume group	OR (95% CI)	p-value
1	Low-volume	1	(reference)
	Mid-volume	1.33 (0.44–4.44)	0.624
	High-volume	0.94 (0.3–3.28)	0.92
2	Low-volume	1	(reference)
	Mid-volume	0.4 (0.13–1.12)	0.087
	High-volume	0.4 (0.14–1.08)	0.072
3	Low-volume	1	(reference)
	Mid-volume	1.01 (0.27–4.22)	p = 0.986
	High-volume	2.28 (0.74–8.71)	p = 0.174
4	Low-volume	1	(reference)
	Mid-volume	1.41 (0.63–3.34)	p = 0.414
	High-volume	0.81 (0.36–1.98)	p = 0.628
5	Low-volume	1	(reference)
	Mid-volume	0.72 (0.3–1.78)	p = 0.461
	High-volume	0.43 (0.17–1.06)	p = 0.056

## Data Availability

The KID database is available through Healthcare Cost & Utilization Project (HCUP) website. Citation: KID Database Documentation. Healthcare Cost and Utilization Project (HCUP). March 2024. Agency for Healthcare Research and Quality, Rockville, MD. www.hcup-us.ahrq.gov/db/nation/kid/kiddbdocumentation.jsp
