# Supplementary material for: Center Volume Not Associated with Survival Benefit of Inter-hospital Transfer for Pediatric CardiacSurger
Source: Res Sq. 2024 Nov 20:rs.3.rs-5356715. Preprint. [Version 1] doi: 10.21203/rs.3.rs-5356715/v1 (PMC11601875; doi:10.21203/rs.3.rs-5356715/v1)
Supplement: Supplement 1 [file NIHPPRS5356715V1-supplement-1.pdf]

Supplementary Files

This is a list of supplementary files associated with this preprint. Click to download.

- SupplementaryappendixKIDtransfermanuscript.docx
